# Cancer Cell Cytotoxicity of Marinopyrroles, Pyrrolomycins, and Their Derivatives

**DOI:** 10.3390/md23100403

**Published:** 2025-10-16

**Authors:** Jeffrey M. Zimmerly, Nicholas A. Armstrong, Clare F. Euteneuer, Brianna N. Davis, M. Beth Griffis-Anchala, Angelique Vargas, Paul H. Davis

**Affiliations:** 1 Department of Biology, University of Nebraska at Omaha, Omaha, NE 68182, USA; jzimmerly@unomaha.edu (J.M.Z.); nicholasarmstrong@unomaha.edu (N.A.A.); ceuteneuer@unomaha.edu (C.F.E.); bnd24@byu.edu (B.N.D.); bgriffis-anchala@unomaha.edu (M.B.G.-A.); angeliquevargas@unomaha.edu (A.V.); 2 College of Medicine, University of Nebraska Medical Center, Omaha, NE 68198, USA; 3 Department of Microbiology and Molecular Biology, Brigham Young University, Provo, UT 84604, USA

**Keywords:** marinopyrrole, pyrrolomycin, anticancer, marine-derived compounds

## Abstract

Marine-derived secondary metabolites have emerged as a rich potential source of anticancer agents, with marinopyrroles and pyrrolomycins representing structurally distinct halogenated pyrroles of interest. Initially characterized for their potent antibacterial properties, these compounds were later shown to exert cytotoxic activity across diverse hematologic and solid malignancies, frequently correlating with Mcl-1 dependence. Marinopyrrole A, a marine-derived natural product, exemplified this potential by inducing proteasomal degradation of Mcl-1, thereby sensitizing resistant cancer cells to Bcl-2 inhibitors and TRAIL-based therapies. In parallel, pyrrolomycins, particularly pyrrolomycin C and members of the F-series, demonstrated potent activity with submicromolar IC_50_ concentrations across multiple cancer cell lines, and also perturbed cytoskeletal and membrane integrity. Together, these halogenated pyrroles illustrate multifaceted cancer cell cytotoxicity profiles but face translational barriers, including mechanistic ambiguity, poor solubility, and off-target toxicities. To address these limitations, extensive medicinal chemistry efforts have yielded synthetic derivatives with improved potency, selectivity, and drug-like properties, with notable examples such as MP1 and KS18 showing enhanced efficacy in MYC-driven neuroblastoma, medulloblastoma, and drug-resistant multiple myeloma.

## 1. Introduction

Cancer remains a leading cause of morbidity and mortality worldwide, with 20 million new cases and 9.7 million deaths in 2022 alone. Lifetime cancer risk remains high: approximately one in five people will develop cancer, and roughly one in nine men and one in twelve women will die of the disease [1]. Based on current projections, by the year 2050, cancer cases are expected to rise by 76.6%, equating to just over 35 million new cases each year [2]. Despite advancements in tumor and molecular biology, many limitations persist in the treatment of cancer. Curative surgery is generally feasible only for early-stage disease, while current radiotherapy and chemotherapy face obstacles of toxicity, drug resistance, and escalating costs [3,4,5,6]. These major limitations in the current paradigm of cancer treatment have resulted in the extensive isolation and study of marine-derived products. Many bioactive molecules derived from marine organisms have been studied for their ability to induce apoptosis in cancer cells, leading to several subsequent clinical trials to test efficacy in human malignancies [7]. Several marine-derived compounds and their analogs have gained FDA approval as anticancer medications, including cytarabine, trabectedin, eribulin mesylate, and lurbinectedin, plus monomethyl auristatin E as a cytotoxic payload of antibody–drug conjugates [8,9,10,11,12]. These successes underscored the marine environment as a notable reservoir of potent anticancer molecules.

Marine-derived *Streptomyces* species are prolific producers of structurally diverse and biologically active natural products, with several gaining FDA approval as anticancer agents, including the anthracyclines daunorubicin, doxorubicin, epirubicin, idarubicin, and valrubicin [13]. They have also been found to produce other potential anticancer compounds, including marinopyrroles and pyrrolomycins [14]. The marinopyrroles A-F are defined by a unique 1,3′-bipyrrole core scaffold, with structural diversity largely arising from differences in halogenation patterns. The first members of this family, marinopyrroles A and B, were discovered in 2008 from *Streptomyces* sp. CNQ-418, a strain isolated from marine sediments collected near La Jolla, California [15]. Additional marinopyrroles, C–F, were reported in 2010 [16]. The pyrrolomycins A–F share close structural resemblance to the single pyrrole half of the marinopyrroles, likewise produced by *Streptomyces* spp. [17,18]. Although these compounds can be isolated from *Streptomyces* spp. broth, yields of natural marinopyrroles and pyrrolomycins are typically in the low milligram per liter range, requiring labor-intensive multi-step extraction and purification [16,19,20]. Consequently, most of the marinopyrroles and pyrrolomycins studied today are obtained via laboratory synthesis. Although total synthesis of these halogenated pyrroles has historically been challenging, recent advances in synthetic strategies have made their preparation increasingly feasible, including a nine-step synthesis of marinopyrrole A that achieved an overall yield of 30% [21,22].

Early investigation of marinopyrroles and pyrrolomycins centered on their antibacterial activity. When tested against Gram-positive bacteria, including methicillin-resistant *Staphylococcus aureus*, marinopyrrole A and B produced MIC_90_ values of 0.31 μM and 1.1 μM, respectively, indicating promising potency [15]. Marinopyrrole A has also been shown to be effective against a subset of Gram-negative bacteria, including *Haemophilus influenzae*, *Neisseria gonorrhoeae*, *Moraxella catarrhalis*, and *Campylobacter jejuni*, each producing 50% inhibitory concentration (IC_50_) values below 1 mg/L [23]. Several naturally occurring pyrrolomycins have also shown efficacy against a broad range of bacterial pathogens, including *Staphylococcus aureus*, *Streptococcus faecalis*, and *Bacillus anthracis* [24]. Although the earliest studies of these compounds emphasized their antibiotic properties, cytotoxicity against cancer cell lines hinted at further therapeutic potential beyond infectious disease [15].

Among naturally occurring compounds, marinopyrrole A, also known as maritoclax, was the first to demonstrate cancer cell cytotoxicity and remains one of the most extensively studied members of the marinopyrrole and pyrrolomycin families. Early on, marinopyrrole A was reported as a selective inhibitor of the anti-apoptotic protein Mcl-1, a validated target for cancer treatment and a likely mediator of its cytotoxic activity [25,26]. Additional preclinical studies demonstrated the cytotoxicity of marinopyrrole A across various tumor cell lines, underscoring its initial promise as an anticancer lead compound. Marinopyrroles B, C, and F, along with pyrrolomycin C and the F-series pyrrolomycins (F1, F2a, F2b, F3), achieve IC_50_ concentrations less than or equal to marinopyrrole A across multiple cancer cell lines [16,21,27].

The promise of naturally occurring marinopyrroles and pyrrolomycins has inspired extensive analogue development. Medicinal chemistry efforts have yielded numerous derivatives, modifying the marinopyrrole and pyrrolomycin scaffold to improve efficacy, minimize toxicity, alter target affinity, and enhance drug-like properties to increase clinical translation efforts of the compound class. For instance, several pyrrolomycin derivatives containing an altered halogenation pattern and nitro-group additions showed increased efficacy against the HCT-116 colon cancer cell line and MCF7 breast cancer cell line compared to the naturally occurring pyrrolomycin C [27]. Additionally, three newly synthesized derivatives also produce greater selectivity indexes than pyrrolomycin C, indicating reduced toxicity of derivatives to healthy cells. Other derivatives, such as MP1, have led to enhanced drug-like properties compared to the naturally occurring marinopyrrole A, including increased solubility [28,29]. The efficacy of MP1 when targeting MYC-amplified medulloblastoma cell lines produced potent IC_50_ values below 1 μM [30]. Such findings underscore the versatility of the marinopyrrole and pyrrolomycin scaffolds and their potential to target distinct malignancies.

Collectively, marinopyrroles, pyrrolomycins, and their derivatives represent a promising class of marine-derived molecules with preclinical efficacy against a broad range of solid and hematologic cancers. This review will summarize their mechanism of action, biologic activity, and ongoing efforts to optimize their therapeutic utility, which largely focuses on reducing off-target effects and increasing solubility.

## 2. Mechanism of Action

### 2.1. Mcl-1 Inhibition and Proteasomal Degradation

The naturally occurring marinopyrroles and pyrrolomycins have primarily been studied for their potential role as BH3-mimetics, compounds that mimic the function of pro-apoptotic BH3-only proteins such as BIM, BID, PUMA, and NOXA. These BH3-only proteins represent a distinct subclass of the Bcl-2 family that contains only the BH3 domain, which is sufficient to initiate pro-apoptotic signaling. Under normal conditions, they serve as critical triggers of apoptosis but are often neutralized by anti-apoptotic proteins, including Mcl-1, Bcl-2, Bcl-xL, and Bcl-W. Many cancers exploit this mechanism by overexpressing anti-apoptotic proteins to evade apoptosis [31,32]. BH3-mimetics restore apoptotic potential by disrupting inhibitory complexes formed when anti-apoptotic proteins sequester BH3-only proteins in their hydrophobic binding grooves. By competitively binding to these grooves, BH3-mimetics release BH3-only proteins, enabling them to activate the pro-apoptotic effectors BAX and BAK. Once activated, BAX and BAK induce mitochondrial outer membrane permeabilization, which releases pro-apoptotic factors and initiates the cascade of events that culminates in apoptotic cell death [33].

**Figure 1:**
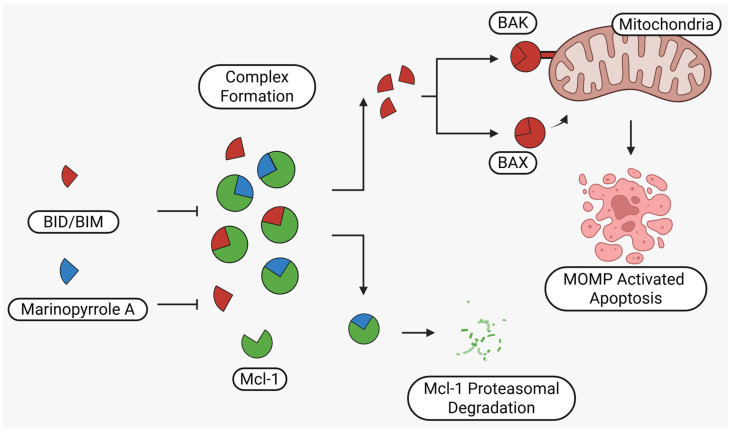
Schematic illustration of the proposed mechanism of action of Marinopyrrole A. Marinopyrrole A binds to the anti-apoptotic protein, myeloid cell leukemia-1 (Mcl-1), promoting its proteasomal degradation. Loss of Mcl-1 releases the BH3-only proteins BID and BIM, which subsequently interact with the pro-apoptotic proteins BAK and BAX. This interaction activates mitochondrial outer membrane permeabilization (MOMP), initiating the intrinsic apoptosis pathway.

Marinopyrrole A interfered with BIM/Mcl-1 binding, as demonstrated by ELISA-based peptide displacement assays (IC_50_ ≈ 10 µM) [34]. NMR spectroscopy using N-labeled Mcl-1 identified chemical shift perturbations near the p4 pocket, suggesting this as the likely ligand-binding region [35]. In cellular contexts, marinopyrrole A often promotes selective proteasomal degradation of Mcl-1 without significantly affecting other anti-apoptotic proteins, including Bcl-2 or Bcl-xL (Figure 1).

The requirement for proteasomal degradation in mediating marinopyrrole A-induced apoptosis was demonstrated in melanoma models, where proteasome inhibition markedly reduced both Mcl-1 degradation and apoptosis [36]. Similar findings in acute myeloid leukemia (AML) showed apoptosis induction directly correlating with Mcl-1 loss, underscoring the therapeutic potential in Mcl-1–dependent malignancies (Figure 1) [35,37].

Marinopyrrole A was explored as a preclinical combination therapy candidate with inhibitors of Bcl-2 family proteins to extend its apoptotic potency. ABT-737 and its orally active analog ABT-263 (navitoclax) are established inhibitors of Bcl-2, Bcl-W, and Bcl-xL, but display weak activity against Mcl-1. Additionally, ABT-199 (venetoclax) specifically targets Bcl-2, with minimal inhibition of other anti-apoptotic proteins [38]. Clinical trials with venetoclax have demonstrated favorable outcomes, including improved progression-free survival in chronic lymphocytic leukemia and small lymphocytic lymphoma [39,40,41,42]. However, resistance may emerge in cancers with elevated Mcl-1 expression, where sequestration of pro-apoptotic proteins such as BIM, PUMA, or NOXA prevents apoptosis. Preclinical studies indicate that co-treatment with marinopyrrole A can overcome this resistance by promoting proteasomal degradation of Mcl-1 and, in some contexts, reduce levels of additional anti-apoptotic proteins [36,43,44,45,46]. This mechanistic complementarity distinguishes marinopyrrole A from classical BH3-mimetics, which act primarily through competitive binding rather than degradation [47,48]. Accordingly, marinopyrrole A may act as a mechanistically distinct partner in combination strategies, expanding the scope of apoptosis induction not only with venetoclax but also potentially with other Bcl-2 family inhibitors.

### 2.2. Integration of Mcl-1 Degradation into Apoptotic Signaling Networks

Marinopyrrole A has shown the capability to induce apoptosis in an Mcl-1-dependent and Mcl-1-independent manner, indicating its ability to interact with multiple pathways [49]. Beyond Mcl-1, it can simultaneously modulate other Bcl-2 family proteins, including Bcl-xL and Bcl-2, indicating that Mcl-1 proteasomal degradation may occur within a broader network of apoptotic regulation [44]. The mechanistic complexity of marinopyrrole A was further underscored in cancers harboring epidermal growth factor receptor (EGFR) mutations and tumors exhibiting elevated phosphorylated AKT (p-AKT). Both oncogenic drivers sustain Mcl-1 by enhancing its transcription and protecting it from proteasomal degradation, thereby reinforcing apoptotic resistance. By promoting Mcl-1 degradation, marinopyrrole A directly counters these survival signals, explaining the heightened sensitivity of EGFR-mutant and p-AKT-high non-small cell lung cancer (NSCLC) cells to Mcl-1 inhibition. Importantly, these cells exhibit strong synergistic responses when Mcl-1 degradation is combined with Bcl-2 or Bcl-xL inhibition, illustrating the networked regulation of apoptotic resistance within the Bcl-2 family [45]. Supporting this view, a series of structure–activity analyses revealed that certain cyclic marinopyrrole A derivatives significantly disrupt the interactions of both Mcl-1 and Bcl-xL with the pro-apoptotic protein BIM, highlighting their dual-targeting potential as BH3-mimetic scaffolds [50].

### 2.3. Actin Cytoskeleton Targeting as a Secondary Mechanism of Marinopyrrole A

Marinopyrrole A has been shown to directly target the actin cytoskeleton through a covalent binding interaction that is distinct from its proposed effects on Bcl-2 family proteins. Using an acyl dye transfer approach, biochemical studies demonstrated that marinopyrrole A selectively and covalently modifies actin at lysine 115, a site spatially separate from ATP-, gelsolin-, and profilin-binding regions as well as the latrunculin A pocket. This interaction occurs with high affinity (Kd ≈ 0.12 ± 0.2 µM) and functionally impairs actin dynamics, as shown by inhibition of pyrene-actin polymerization in vitro (IC_50_ of 39.5 ± 6.2 µM). Because pyrene-actin polymerization is a widely used surrogate for filament assembly [51], this inhibition provides direct evidence that marinopyrrole A binding disrupts actin filament formation, linking covalent modification to cytoskeletal dysfunction. In live HCT-116 cells, probe-labeled marinopyrrole A co-localized with F-actin filaments and induced marked cytoskeletal disruption, correlating with growth inhibition at low micromolar concentrations. Additionally, it has been shown that F-series pyrrolomycins impair biological membranes and induce cytoskeletal rearrangements, triggering non-canonical cell death mechanisms, notably autophagy and methuosis [21]. Subsequent experiments using latrunculin A, a known actin polymerization inhibitor, found that comparable cytoskeletal disruption alone did not trigger apoptosis, suggesting that actin modulation by marinopyrrole A is not sufficient to account for its full cytotoxic profile [52,53]. This supports the view that actin targeting could function as a secondary mechanism, contributing to phenotypic outcomes in concert with other cellular pathways rather than serving as the primary driver of cell death.

### 2.4. Mitochondrial Dysfunction and ROS Accumulation

Emerging evidence indicates that marinopyrrole A exerts additional cytotoxic effects through disruption of mitochondrial integrity, representing a secondary mechanism distinct from its action on Mcl-1. Treatment induces pronounced mitochondrial fragmentation and the accumulation of reactive oxygen species (ROS), accompanied by disruptions in the electron transport chain function, particularly at complexes I and III [54]. These complexes are recognized as principal sites of mitochondrial ROS generation, with complex III-derived ROS playing central roles in both stress signaling and apoptotic pathways [55]. Comparable to the target inhibition of these complexes, marinopyrrole A exposure results in ROS accumulation, adenosine triphosphate (ATP) depletion, and cell death features consistent with mitochondrial dysfunction [56,57]. Notably, these effects occur largely independently of Mcl-1 status but appear dependent on Bax/Bak-mediated mitochondrial outer membrane permeabilization [58]. Although ROS accumulation alone is insufficient to trigger apoptosis, its contribution to mitochondrial stress likely amplifies marinopyrrole A’s broader cytotoxic profile.

### 2.5. TRAIL Sensitization via DR5 and cFLIP Regulation

Beyond direct Mcl-1 inhibition, recent findings demonstrated that marinopyrrole A has also been shown to sensitize cancer cells to tumor necrosis factor-related apoptosis-inducing ligand (TRAIL)-mediated apoptosis through a dual regulatory mechanism. First, marinopyrrole A induced the C/EBP homologous protein (CHOP), a transcriptional factor activated during endoplasmic reticulum stress, which in turn drives transcriptional upregulation of death receptor 5 (DR5). Elevated DR5 expression increases surface receptor availability and enhances assembly of the death-inducing signaling complex (DISC), thereby augmenting susceptibility to TRAIL-induced caspase activation [59]. Second, marinopyrrole A upregulates the microRNA miR-708, which directly targets and downregulates cellular FLICE-inhibitory protein (cFLIP). As a negative regulator of apoptosis, cFLIP competes with caspase-8 for binding at the DISC; its suppression permits efficient caspase-8 activation and apoptotic progression [60]. Collectively, these findings establish a CHOP-DR5 and miR-708-cFLIP axis as a mechanistic basis by which marinopyrrole A lowers the apoptotic threshold and potentiates TRAIL responsiveness in leukemia cells.

### 2.6. MYCN/MYC Modulation with MP1

The MYC family of oncogenes, including MYC and MYCN, are among the most potent drivers of oncogenesis and are implicated in aggressive tumor subtypes across both pediatric and adult cancers. Their amplification or overexpression promotes uncontrolled proliferation, metabolic reprogramming, and therapy resistance, making them critical but historically not targetable in cancer therapy [61,62].

Recent studies have established that the marinopyrrole A derivative MP1 exerts direct mechanistic effects on the MYC family of oncogenes, particularly MYCN in neuroblastoma and MYC in medulloblastoma. In MYCN-amplified neuroblastoma, MP1 treatment led to marked destabilization of MYCN protein, accompanied by decreased Mcl-1 expression and induction of autophagic flux, as reflected by increased LC3II accumulation, a widely used marker of autophagosome formation and autophagic activity [28,63]. These molecular changes were coupled to mitochondrial disruption, including cristae loss and altered morphology, as well as a collapse in oxidative phosphorylation and glycolytic capacity, driving cells toward a quiescent state [28,30]. Together, these findings demonstrate that MP1 simultaneously undermines both oncogenic MYCN signaling and core metabolic integrity.

In MYC-driven medulloblastoma, MP1 similarly suppressed growth through destabilization of MYC protein, but the downstream effects diverged in notable ways. Transcriptomic profiling showed that MP1 treatment was associated with significant and broad MYC-associated transcriptional programs, including pathways governing translation initiation and mTOR signaling [30]. This repression extended to MYC-dependent metabolic pathways, leading to reduced global protein synthesis and energy depletion. Mechanistically, MP1 shortened MYC protein half-life, thereby destabilizing oncogenic transcriptional output and reinforcing cell cycle arrest and apoptosis [30]. Unlike in neuroblastoma, where mitochondrial morphology and autophagy predominated as mechanistic readouts, the medulloblastoma context emphasized transcriptome-level reprogramming and suppression of anabolic growth pathways.

Taken together, these studies identify MYCN/MYC destabilization as a distinctive mechanistic feature of marinopyrrole A derivatives. Downstream mechanistic pathways diverge, manifesting as metabolic and mitochondrial collapse in MYCN-amplified neuroblastoma, whereas transcriptional and translational repression predominates in MYC family-driven medulloblastoma. Despite these differences, both outcomes appear to stem from disruption of MYC/MYCN stability. Collectively, the evidence indicates that MP1 disrupts MYC family-driven oncogenic programs through destabilization of MYC and MYCN protein stability. This dual mechanistic framework highlights a previously unrecognized capacity of marinopyrrole derivatives to directly destabilize MYC/MYCN and thereby dismantle their downstream survival networks.

## 3. Natural Compounds

### 3.1. Cancer Cell Cytotoxicity of Natural Marinopyrroles and Pyrrolomycins

Marinopyrrole A, when isolated from its natural source, marine-derived streptomyces species, occurs exclusively as the (−) enantiomer. However, during laboratory synthesis of marinopyrrole A, racemization at elevated temperatures produces a mixture of (+) and (−) enantiomers [15]. These enantiomers demonstrate similar properties, including cytotoxicity to the colon cancer cell line HCT-116 with IC_50_ values of 9.4 μM and 8.8 μM for (+) and (−) enantiomers, respectively [15]. Additionally, binding inhibition of Mcl-1 to BIM was found to be similar between enantiomers and the racemic mixture [50]. These results have led studies to use enantiomers or racemic mixtures of marinopyrrole A interchangeably. Despite their interchangeable use throughout the literature, the (−) enantiomer is the naturally occurring configuration and may exhibit distinct properties from the (+) enantiomer beyond the in vitro assays examined.

Of naturally occurring marinopyrroles and pyrrolomycins, marinopyrrole A was the first to be characterized for cancer cell cytotoxicity, demonstrating preferential toxicity toward cancer cells with elevated Mcl-1 expression compared to healthy cells [43]. Although marinopyrrole A remains the most well-studied natural compound in preclinical research, it has not yet progressed to clinical trials.

In hematologic malignancies, marinopyrrole A has been shown to be effective against a broad range of cell lines and patient isolates, generally displaying low-micromolar efficacy in vitro. Acute myeloid leukemia (AML) cell lines, including those that harbored resistance to ABT-737, were sensitive to (+/−) marinopyrrole A treatment with IC_50_ values identified between 1.4 μM and 7.7 μM [46]. In human patient AML isolates, susceptibility to (+/−) marinopyrrole A was positively correlated with Mcl-1 expression, with those most sensitive displaying IC_50_ values below 10 μM [46]. Similar results were observed in large granular lymphocytic leukemia (LGLL) patient samples, where (+/−) marinopyrrole A potency was positively correlated with Mcl-1 expression: patient samples with the highest Mcl-1 protein levels produced IC_50_ values between 4.64 μM and 11.84 μM [43]. In the relapsed acute lymphoblastic leukemia (ALL) cell line RS4;1, (−) marinopyrrole A treatment produced an IC_50_ value of 2 μM [49]. Multiple myeloma cell line subtypes showed variable sensitivity to (+/−) marinopyrrole A with IC_50_ values between 0.7 μM and 11.1 μM [64]. Across these cell lines, the efficacy of (+/−) marinopyrrole A was correlated with high Mcl-1 expression and low Bcl-2 expression, suggesting a Bcl-2 protein expression profile dependence of the compound [64].

Marinopyrrole A’s single-agent activity has also been tested in solid malignancies, with similar potency seen in the blood cancers. In the triple negative breast cancer cell line MDA-MB-468, (+/−) marinopyrrole A inhibited growth with an estimated IC_50_ value of 2 μM [65]. The treated breast cancer cell line had markedly reduced Mcl-1 levels and increased cleaved caspase 3, signaling the induction of apoptosis [65]. Across three distinct metastatic melanoma cell lines, (+/−) marinopyrrole A induced apoptosis, with cytotoxicity IC_50_ values ranging from 2.2 μM to 5.0 μM [36]. Nasopharyngeal carcinoma (NPC) cell lines HK1 and C666-1 were susceptible to marinopyrrole A growth inhibition with IC_50_ values of approximately 1 μM. Marinopyrrole A was also tested against NPC spheroids, resulting in increased spheroid cell death without acquired resistance to treatment [44]. The cervical cancer cell line HeLa was only modestly sensitive to (−) marinopyrrole A (IC_50_ = 20 μM) despite the Mcl-1 dependence observed in HeLa cells [49]. Marinopyrrole A treatment against a panel of non-small cell lung cancer (NSCLC) cell lines with diverse driver mutations produced IC_50_ values of 1.1 μM to 9.2 μM. Sensitivity to marinopyrrole A was independent of driver mutations and BCL-2 family expression in tested NSCLC lines [45]. In the colon cancer cell line HCT-116, (+) marinopyrrole A and (−) marinopyrrole A inhibited cancer cell growth with IC_50_ values of approximately 9 μM [15].

The only in vivo study of (+/−) marinopyrrole A’s anticancer efficacy was published by Doi et al. in 2014 [46]. Female athymic nude mice bearing U937 AML xenografts were treated daily with intraperitoneal injections of (+/−) marinopyrrole A (20 mg/kg/day) for 28 days. Treated mice showed significantly reduced tumor volumes compared to vehicle alone at 17 days post termination of treatment, with 59.1% of mice demonstrating a greater than 50% reduction in tumor volume. Importantly, no significant toxicities were observed, including the absence of depleted blood cell populations, nor histopathological abnormalities in the liver, kidneys, brain, spleen, and heart. Daily intraperitoneal injections of (+/−) marinopyrrole A produced a maximum tolerated dose (MTD) and 50% lethal dose (LD_50_) of 20 mg/kg/day and 25 mg/kg/day, respectively, in 6-week-old female athymic nude mice.

While marinopyrrole A demonstrates potent single-agent activity across a diverse range of cancer models, increasing attention has been directed towards its use in combination approaches. Marinopyrrole A was shown to act synergistically with other anticancer agents: in several studies, marinopyrrole A functions as a sensitizing agent, overcoming resistance mechanisms to other therapies. These combinations align with the broader therapeutic strategy in oncology, where multi-drug regimens are frequently used to address resistance, improve efficacy, and minimize toxicity to healthy cells. The senolytic compound ABT-737 and its orally active analog ABT-263 (navitoclax) have been commonly studied as a co-treatment with marinopyrrole A. Combination therapy of ABT-737/ABT-263 in ABT-737-resistant hematologic cancer cell lines with sub-optimal doses of (+/−) marinopyrrole A restored sensitivity and synergistically induced apoptosis with co-treatment [43,46]. In UACC903 melanoma cells, co-treatment with (+/−) marinopyrrole A and ABT-737 significantly increased apoptosis [36]. Similarly, combining marinopyrrole A with ABT-263 produced synergistic effects in both 2D and 3D models of NPC, and in 6 out of 9 tested non-small cell lung cancer cell lines [44,45].

Other combination therapies with marinopyrrole A have been explored, including co-treatment with tumor necrosis factor-related apoptosis-inducing ligand (TRAIL). TRAIL primarily binds to the death receptors DR4 and DR5 on the surface of tumor cells, triggering apoptosis^62^. Although clinical trials targeting TRAIL have shown moderate benefit, their efficacy has been limited by widespread resistance mechanisms [66]. Jeon et al. examined the co-treatment of TRAIL and marinopyrrole A in human renal carcinoma, lung cancer, and hepatocellular carcinoma cell lines [59]. Neither 2 µM marinopyrrole A nor 50 ng/mL TRAIL alone significantly induced apoptosis in these cell lines. However, their combination markedly enhanced apoptotic markers, effectively overcoming resistance to TRAIL.

The other naturally occurring marinopyrroles B, C, and F have been tested for cancer cell cytotoxicity against the colon cancer cell line HCT-116. In comparison to marinopyrrole A, marinopyrrole B and C differ by halogenation pattern, with marinopyrrole B containing one additional bromine atom and marinopyrrole C containing one additional chlorine atom. Marinopyrrole B and C against the HCT-116 cell line produced IC_50_ values of 5.3 ug/mL (9.0 μM) and 0.21 ug/mL (0.39 μM) respectively [16]. Marinopyrrole F structurally differs from marinopyrrole A in that it contains an eight-membered ether ring formed through O-aryl cyclization of one salicyloyl arm of marinopyrrole A, accompanied by the loss of a single chlorine atom. Marinopyrrole F produced an IC_50_ of 2.9 ug/mL (6.1 μM) against the HCT-116 cell line [16].

Several naturally occurring pyrrolomycins, including pyrrolomycin C and the pyrrolomycin F-series (F_1_, F_2a_, F_2b_, and F_3_), demonstrated potent in vitro cancer cell cytotoxic activity with sub-micromolar to low-micromolar IC_50_ values against various cancer cell lines. These pyrrolomycins structurally resemble a portion of the marinopyrrole A molecule, with halogenation patterns varying in arrangement and number of bromine and chlorine atoms. Pyrrolomycin C tested against the colon cancer cell line HCT-116 and the breast cancer cell line MCF7 produced IC_50_ values of 0.8 μM and 1.5 μM, respectively [27]. The pyrrolomycin F-series produced IC_50_ values against the HCT-116 and MCF7 cell lines ranging from 0.35 μM to 1.21 μM [21].

In total, marinopyrrole A continues to be the most extensively studied naturally occurring compound of its class, with single-agent efficacy primarily correlated to Mcl-1 expression. However, in some cell lines, other anti-apoptotic Bcl-2 proteins may have meaningful contributions to efficacy. The activity of marinopyrrole A has been demonstrated across hematologic and solid malignancies with roughly equivalent potency, and cytotoxicity against established cell lines and primary patient samples are comparable. Beyond direct cytotoxicity, marinopyrrole A acts as a sensitizing agent when combined with TRAIL or Bcl-2 inhibitors, producing synergistic interactions. While marinopyrrole A continues to dominate investigation, efforts have also extended to other naturally occurring marinopyrroles and pyrrolomycins for their potential anticancer properties. Table 1 provides summaries of the naturally occurring marinopyrroles and pyrrolomycins that have been cited in cancer studies.

### 3.2. Limitations of Natural Marinopyrroles and Pyrrolomycins

#### 3.2.1. Chemical and Structural Limitations

Naturally occurring marinopyrroles and pyrrolomycins have faced several roadblocks preventing their progression into clinical trials, largely stemming from a combination of mechanistic ambiguity, limited potency, toxicity, and pharmacologic issues. Although marinopyrrole A was initially reported as a selective Mcl-1 inhibitor, disrupting the interaction of BIM to Mcl-1, several subsequent studies showed variable targeting of the anti-apoptotic Bcl-2 family, calling into question the true mechanism of the compound [43,49]. Even as a Mcl-1 inhibitor, marinopyrrole A’s binding disruption of BIM to Mcl-1 was only modest, with IC_50_ values of approximately 10 μM, compared to other Mcl-1 targeting compounds such as AZD5991, demonstrating high affinity for Mcl-1 [43,67,68].

Marinopyrrole A’s lipophilicity is thought to mediate its binding to the hydrophobic BH3 domain of Mcl-1; however, this property also poses challenges for drug development. Its calculated logP (ClogP) of 5.6 exceeds Lipinski’s recommended threshold, placing it outside the range of ~90% of orally active molecules. According to Lipinski’s Rule of Five, compounds with ClogP values greater than 5 are more likely to exhibit poor solubility, absorption, and permeability [50,69]. Moreover, studies examining marinopyrrole A against bacterial and parasitic pathogens in either bovine or human serum in vitro have shown near complete inactivation of compound efficacy, suggesting a high degree of protein binding, greatly reducing its clinical applicability [70,71]. However, multiple in vivo studies using marinopyrrole A have demonstrated efficacy of the molecule in rodent models, suggesting that the serum inactivation may not be complete or not represented accurately within in vitro models [46,71].

#### 3.2.2. Potential Toxicity

Recent work has demonstrated that some marinopyrroles and pyrrolomycins appear to function as protonophores, depolarizing membranes and dissipating the proton-motive force in prokaryotic organisms [72,73]. Protonophores, such as CCCP and FCCP, perturb the proton-motive force within the mitochondrial membrane, leading to ATP depletion and cell death in eukaryotic cells [74]. This potential protonophore activity of some natural marinopyrroles and pyrrolomycins has raised concern for potential mitochondrial toxicity of the molecules. The possible targeting of Mcl-1 also leads to concerns of potential cardiovascular adverse effects, which are common issues across the spectrum of Mcl-1 inhibitors under active investigation [75].

Despite considerable concerns regarding the toxicity of natural marinopyrroles and pyrrolomycins, very little in vivo safety data has been reported to date. Beyond a single xenograft study of marinopyrrole A showing decreased tumor-burden when treated at the MTD, there is a stark absence of in vivo data regarding efficacy and pharmacokinetic/pharmacodynamic properties for all naturally occurring marinopyrroles. Additionally, no naturally occurring pyrrolomycin has ever been evaluated in tumor bearing mice. This deficiency of in vivo validation raises significant questions about the therapeutic window and clinical translation of these compounds as anticancer agents.

Together, these barriers, including the disputed mechanism of action, potential limited potency in serum, unfavorable lipophilicity, and potential toxicities, have given naturally occurring compounds a more difficult path to clinical use. These challenges have ushered in the synthesis of novel derivatives to address these hurdles.

## 4. Synthetic Derivatives

### Anti-Cancer Activity of Synthetic Derivatives

Despite the promise of naturally occurring marinopyrroles and pyrrolomycins, their limitations have inspired a plethora of synthetic derivatives. Resulting analogs exhibit a variety of enhanced characteristics, including differential activity against protein–protein interactions of the Bcl-2 family, increased potency against varying cancer cell lines and patient samples, increased solubility, and minimized toxicity. Table 2 provides summaries of some of the analogs that have been cited in the cancer literature.

Cheng et al. synthesized symmetrical and cyclic marinopyrrole analogs and evaluated their inhibitory effects on binding between anti- and pro-apoptotic proteins [50]. Among symmetrical derivatives, compound **9**, a symmetrical tetrabrominated analog of marinopyrrole A, demonstrated increased potency against the Bcl-2 family with IC_50_ values of 4.5 µM for Mcl-1/Bim and 7.3 µM for Bcl-xL/Bim, a ~2-fold improvement over marinopyrrole A. Compound **9** also induced caspase 3 cleavage in the cancer cell line MDA-MB-468, indicating the induction of apoptosis. Two cyclic analogs, compounds **3** and **4**, bearing OSO_2_CF_3_ and COOMe substituents, respectively, exhibited further increased activity with an approximate 5-fold improvement over marinopyrrole A. Compound **4** also produced an increased solubility, with a ClogP value of 4.7. Despite some cyclic analogs showing improved targeting of the Bcl-2 family, none produced meaningful cancer cell cytotoxicity in intact cancer cell lines. Additionally, the toxicity of these analogs has not been evaluated, leaving unanswered questions regarding their therapeutic window.

A subsequent study synthesized symmetrical marinopyrrole derivatives with sulfide and sulfone spacers substituted in the para-position relative to the carbonyl group of both aromatic rings. Cheng et al. reported that sulfide-linked derivatives, particularly benzyl- and benzyl methoxy-substituted compounds **4** and **5**, were potent dual inhibitors with IC_50_ values of 0.6–0.7 µM for both Mcl-1/Bim and Bcl-xL/Bim [65]. Compounds **4** and **5** exhibited a 12.7-fold increase in Mcl-1/Bim binding inhibition and a 27.3-fold increase in Bcl-XL/Bim binding inhibition compared to marinopyrrole A. Despite their increased anti-apoptotic Bcl-2 family inhibition, efficacy against the breast cancer cell line MDA-MB-468 was limited. This limited efficacy against intact cancer cells may stem from the compound’s high lipophilicity, with ClogP values above 9. Additional derivatives 3 and 11 had reduced selectivity for the Bcl-2 family but produced superior growth inhibition against the breast cancer cell line with IC_50_ values of 2–3 μM. Derivatives 3 and 11 produced more favorable ClogP values of 6.1 and 4.5, respectively. Together, these data may suggest a trade-off: while adding hydrophobic substituents can increase lipophilicity and improve binding within the BH3 hydrophobic groove, excessive lipophilicity may compromise the drug-like properties necessary for efficacy in intact cells. While these derivatives highlight the ability to manipulate the marinopyrrole scaffold to alter biological activity, toxicity remains unexplored.

In a structure–activity relationship (SAR)-driven study, Li et al. synthesized a library of marinopyrrole derivatives, notably incorporating bistriazole spacers, sulfide spacers, and large hydrophobic groups to improve interactions within the p1 and p4 pockets of Mcl-1 [76]. Compound **42**, which includes an octyl group, exhibited IC_50_ values for Mcl-1/Bim and Bcl-XL/Bim binding inhibition of 0.6 μM and 0.5 μM, respectively. These results identify compound **42** as the most potent dual inhibitor marinopyrrole derivative to date. Selectivity profiling also identified compounds **34** and **36** with 16-fold and 13-fold preference for Mcl-1 over Bcl-xL. Mechanistic studies using fluorescence quenching and NMR supported direct binding of these analogs to Mcl-1 at the BIM BH3 groove. No toxicity data were reported for these derivatives.

McGuire et al. evaluated the efficacy of the novel pyrrolomycin analog MP1 in MYCN-amplified, chemo-resistant neuroblastoma cell lines [28]. MP1 possesses greatly increased solubility with a ClogP value of 3.8. MYCN amplification, a hallmark of high-risk neuroblastoma, was positively correlated with sensitivity to MP1 across tested cell lines. The BE(2)-C cell line, characterized by high MYCN expression, exhibited the greatest sensitivity, with an IC_50_ of 0.096 µM. Co-treatment of BE(2)-C cells with MP1 and temsirolimus (TEM), an FDA-approved mTOR inhibitor used to treat renal cell carcinoma and has also been investigated in neuroblastoma treatment, produced a synergistic effect, lowering the IC_50_ value to 0.023 µM. To assess in vivo efficacy and tissue distribution, NSG mice bearing subcutaneous BE(2)-C neuroblastoma xenografts were administered MP1 orally at 15 mg/kg/day, five days per week, with 10 total doses given. MP1 successfully penetrated tumor tissue, reaching concentrations above its in vitro IC_50_. Additionally, MP1 was well tolerated, including bone marrow cells, at the given dosing strategy. In a follow-up experiment, mice received daily treatment with MP1 (15 mg/kg, oral), TEM (10 mg/kg, intraperitoneal), or the combination for five days per week with 10 total doses. The combination therapy exhibited the slowest tumor growth among the groups, with a trend toward statistical significance (*p* = 0.06).

Building on these findings, Coulter et al. extended the evaluation of MP1 to medulloblastoma, focusing on MYC-amplified subgroups where MYC acts as a functional homologue of MYCN [30]. Consistent with the neuroblastoma data, medulloblastoma cell lines with elevated MYC expression were significantly more responsive to MP1. Furthermore, co-treatment with TEM enhanced MP1 activity in MYC-amplified medulloblastoma cell lines, suggesting a synergistic interaction. Synergy between MP1 and TEM was confirmed in NSG mice bearing subcutaneous xenografts of MYC-amplified medulloblastoma HD-MB03 cells. Mice received MP1 (10 mg/kg, oral, 5×/week), TEM (10 mg/kg, intraperitoneal, 3×/week), or combination treatment for 21 days. MP1 treatment alone reduced tumor volume by approximately 50% compared to vehicle alone. The combination treatment of MP1 and TEM further reduced tumor volume by 60% compared to MP1 alone. In a follow-up experiment, orthotopic xenografts were established via intracerebellar injection of HD-MB03 cells. Ten days post-implantation, mice received the same treatment regimens described previously. Kaplan–Meier survival analysis showed that the combination therapy of MP1 and TEM significantly prolonged survival compared to vehicle alone or each monotherapy. Quantification of H&E-stained cerebellar sections demonstrated the largest reduction in brain tumor burden in the combination treatment group.

Lotfy et al. took a novel approach to anticancer drug design by integrating spirooxindole-based MDM2 inhibitors with a marinopyrrole A-inspired pyrrole core via a carbonyl spacer [77]. This hybrid structure aimed to combine MDM2 inhibition, which promotes p53-mediated apoptosis in wild-type p53 cancers, with the BCL2-modulatory properties of marinopyrrole A. Among the synthesized compounds, **5i** and **5q** emerged as the most promising, exhibiting potent pro-apoptotic activity with IC_50_ values below 1µM in MDA-MB, HepG2, and Caco-2 cancer cell lines, adequate solubility, and minimal toxicity to healthy cells. 100% effective concentration (EC_100_) values were calculated against the healthy human fibroblast cell line Wi-38 to assess toxicity profiles of the novel analogs. EC_100_ values for **5i** and **5q** were identified as 2.293 μM and 0.058 μM, respectively, indicating the potential for a usable therapeutic window. Structurally, **5i** features a hexahydropyrrolizine spiro ring system with a 5-chlorosubstituted core and a terminal 4-chlorophenyl group. In contrast, **5q** incorporates a decahydropyrroloisoindole spiro ring, 5-chloro substitution on the indolinone core, and a terminal 3-fluorophenyl ring. Additionally, they demonstrated high-affinity binding to MDM2 (K_D_ = 1.32 µM and 1.72 µM) and significantly downregulated BCL2 gene and protein expression in cancer cells, reinforcing their mechanism of inducing apoptosis through dual targeting of the p53 and BCL2 pathways.

Pyoluteorin derivatives inspired by marinopyrrole A have been synthesized and evaluated for activity against hematological malignancies [35,78]. These derivatives possess roughly half the molecular weight of marinopyrrole A and share structural similarity with pyrrolomycins. Doi et al. synthesized over 30 derivatives, including KS01–KS31, each containing a single pyrrole moiety [35]. Among these, KS04 and KS18 emerged as promising leads. KS04 demonstrated cytotoxic potency comparable to marinopyrrole A across a panel of cancer cell lines, while KS18 consistently outperformed it. However, KS18 was more toxic to mouse primary bone marrow cells compared to KS04, with approximate IC_50_ values of 2 μM and 7 μM, respectively. Both KS18 and KS04 were less toxic to these primary bone marrow cells compared to daunorubicin, a commonly used FDA-approved chemotherapeutic prescribed for the treatment of hematologic malignancies. NMR spectroscopy and docking studies confirmed that KS04 and KS18 directly bind Mcl-1 at the BH3-binding groove, reducing Mcl-1 protein half-life from 178 min to 21 and 20 min, respectively. Both compounds induced apoptosis selectively in Mcl-1–dependent cells and synergized with ABT-737 to enhance apoptosis at sub-optimal doses. In vivo, KS18 was evaluated in nude mice bearing ABT-737–resistant HL-60 xenografts. Daily intraperitoneal administration of KS18 (10 mg/kg/day) alone or in combination with ABT-737 (20 mg/kg/day) for 14 consecutive days significantly reduced tumor volume, with combination treatment producing the greatest reduction in tumor volume. The intraperitoneal MTD and LD_50_ of KS18 were determined to be 10 mg/kg/day and 15 mg/kg/day, respectively. The oral MTD and LD_50_ of KS18 were observed at 20 mg/kg/day and 30 mg/kg/day, respectively.

Al-Odat et al. investigated KS18 in multiple myeloma (MM), where apoptosis was shown to depend on Mcl-1 expression [78]. MM cell lines resistant to common chemotherapies, including venetoclax, ABT-737, bortezomib, and lenalidomide, were more sensitive to KS18 than their non-resistant counterparts. These resistant lines consistently exhibited elevated Mcl-1 expression relative to other anti-apoptotic proteins, supporting previous literature showing Mcl-1 upregulation may be a mediator of drug resistance in MM [79]. KS18 also demonstrated greater cancer cell death than the Bcl-2/Bcl-xL inhibitors venetoclax and ABT-737, further highlighting Mcl-1 as a dominant survival factor in resistant MM. Additionally, KS18 synergized with FDA-approved chemotherapeutic agents, including cyclophosphamide/dexamethasone and doxorubicin/dexamethasone in bortezomib-resistant cell lines. In vivo efficacy was confirmed using U266 MM xenografts in NSG mice treated weekly with intraperitoneal KS18 (5 or 10 mg/kg) for four weeks. KS18 reduced mean tumor volume by approximately 95% compared to controls, with minimal observed toxicity.

## 5. Discussion

Natural marinopyrroles and pyrrolomycins represent a unique class of marine-derived secondary metabolites with intriguing cancer cell cytotoxicity, but this activity has been largely limited to in vitro assays. Among them, marinopyrrole A has been the most extensively characterized, demonstrating low-micromolar potency across diverse hematologic and solid cancer models [43]. Importantly, sensitivity to marinopyrrole A has often correlated with Mcl-1 expression, giving the molecule potential in Mcl-1-dependent malignancies. Single-agent efficacy in xenograft models, coupled with the absence of overt toxicities, further supports its potential clinical relevance. At the same time, combination strategies, particularly with Bcl-2/Bcl-xL inhibitors and TRAIL-based regimens, have highlighted its ability to overcome resistance mechanisms, implicating the potential use of marinopyrrole A as a sensitizer within multi-drug approaches.

Despite these encouraging findings, clinical translation remains uncertain. Foremost are the uncertainties surrounding marinopyrrole A’s mechanism of action and broader toxicity profile. While initially reported as a selective Mcl-1 inhibitor, subsequent studies have demonstrated variable targeting across the Bcl-2 family [49,54]. This ambiguity complicates efforts to define therapeutic windows and raises concerns regarding off-target effects. Equally problematic are pharmacologic limitations: Marinopyrrole A’s high lipophilicity (ClogP = 5.6) reduces drug-likeness, while serum-binding studies may suggest loss of activity in biological fluids. The discovery that these compounds can function as protonophores, dissipating the proton-motive force in prokaryotic and potentially eukaryotic cells, raises the possibility of mitochondrial and cardiac toxicities [73,80]. Collectively, these limitations underscore why natural marinopyrroles and pyrrolomycins have not progressed into clinical evaluation, despite robust in vitro and preclinical efficacy.

To address these limitations, a range of synthetic derivatives have been developed. These analogs have successfully improved upon key weaknesses, including potency, selectivity, and solubility. SAR studies have identified derivatives with sub-micromolar IC_50_ values against Mcl-1/BIM and Bcl-xL/BIM interactions, in some cases achieving 10-to-20-fold greater activity than the parent compound [50,76]. Importantly, certain analogs such as MP1 have also demonstrated strong activity in high-risk neuroblastoma and medulloblastoma models, with in vivo evidence of tumor penetration, reduced growth, and enhanced survival when combined with temsirolimus. Similarly, pyoluteorin-derived analogs such as KS18 have shown remarkable efficacy in xenograft models of drug-resistant multiple myeloma, reinforcing the therapeutic relevance of Mcl-1 inhibition in resistant cancers. These advances highlighted how rational design has transformed the scaffold of marinopyrrole A into more drug-like candidates with greater translation potential.

Mechanistic studies have further advanced the therapeutic relevance of marinopyrroles and pyrrolomycins. Marinopyrrole A not only induces proteasomal degradation of Mcl-1 but also modulates broader apoptotic networks, including Bcl-2 and Bcl-xL, and interactions with signaling pathways defined by EGFR and AKT [43,45]. In addition, evidence for secondary mechanisms, including actin cytoskeletal disruptions [53], mitochondrial dysfunction with ROS accumulation [54], and sensitization to TRAIL via DR5 upregulation and cFLIP downregulation [59], suggests a multifaceted cytotoxic profile. While diverse cytotoxic activities may enhance efficacy, they also complicate the interpretation of mechanistic specificity and highlight the need for caution in clinical contexts where off-target toxicities could be limiting.

Taken together, the therapeutic implications of these findings are twofold. First, marinopyrrole A and its derivatives validate Mcl-1 as an actionable vulnerability, particularly in hematological malignancies and solid tumors resistant to other BH3-mimetics. Second, the capacity to synergize with existing therapies, such as venetoclax or TRAIL-based agents, positions this class as promising candidates for combination regimens. However, the limitations of natural products, including disputed mechanisms of action, poor drug-like properties, and toxicity risks, emphasize that these compounds should be viewed as valuable chemical probes and starting points for medicinal chemistry rather than established therapeutic leads. Clinical success will likely depend on derivatives with improved selectivity, a robust pharmacokinetic profile, and above all, reproducible safety in rigorous in vivo models.

## 6. Conclusions

Marinopyrroles and pyrrolomycins have progressed from intriguing marine-derived metabolites to preclinical anticancer leads of interest, though their ultimate therapeutic value remains uncertain. Among them, marinopyrrole A has shown notable activity across hematologic and solid tumor models, often in correlation with Mcl-1 dependence. However, significant unknowns remain, including mechanistic ambiguity, pharmacologic limitations, and toxicities hindering clinical translation. To improve clinical utility, medicinal chemistry efforts have generated derivatives with superior potency, solubility, and selectivity. These analogs, such as MP1 and KS18, highlight the ability of this scaffold to address resistance mechanisms and broaden therapeutic relevance, particularly when combined with existing agents such as venetoclax or TRAIL-based therapies. Still, long-term relevance will depend on rigorous in vivo validation, careful toxicity profiling, and demonstration of durable therapeutic benefit.

### Future Directions

Looking forward, the therapeutic significance of marinopyrrole- and pyrrolomycin-inspired compounds lies not only in their capacity to target Mcl-1, but also in their prospective use as sensitizers within multi-drug regimens. Continued progress will depend on resolving mechanistic uncertainties, optimizing synthesis, reducing off-target effects, pharmacokinetic and toxicity profiles, and developing biomarkers to guide patient selection. For example, future derivatives may be synthesized utilizing metal complexes to improve drug-like properties of the marinopyrrole and pyrrolomycin scaffold. With these advances, marinopyrrole- and pyrrolomycin-based therapeutics have the potential to emerge as clinically actionable agents, offering new strategies to overcome resistance and improve outcomes in oncology.

## Figures and Tables

**Table 1:** Natural Marinopyrroles and Pyrrolomycins.

Compound	Target Cell	Bioactivity	Mechanism of Action	IC_50_ (μM)	Toxicity to Healthy Cells	Reference
Marinopyrrole A 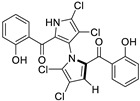	Primary large-granular lymphocyte leukemia, myeloid leukemia (K562), Burkitt’s lymphoma (Raji), acute promyelocytic leukemia (HL60/VCR)	Induces apoptosis in Mcl-1-dependent cell lines, synergistically sensitizes cancer cells to ABT-737 treatment.	Blocks binding of Bim BH3 alpha-helix to Mcl-1, caspase 3 activation, PARP cleavage, proteasomal degradation of Mcl-1, cytochrome c release.	Sensitive LGLL patient samples = 4.64–11.82 μM Raji cells = 2.5 μM marinopyrrole A + 0.051 μM ABT-737	Healthy donor PBMCs were sensitive with IC_50_ = 40–50 μM Less toxicity than pan Bcl-2 inhibitor obatoclax	[43]
	Cervical cancer (HeLa), acute lymphoid leukemia (RS4;11), murine leukemia.	Increased cytotoxicity in Bcl-2-dependent cells compared to Mcl-1-dependent cells.	ND	Hela cells = 20 μM RS4;11 cells = 2 μM	ND	[49]
	Acute myeloid leukemia (HL60, HL60/ABTR, Kasumi-1, Kasumi-1/ABTR, KG1, KG1/ABTR, KG1a/ABTR, U937)	Induces apoptosis, sensitivity correlated to Mcl-1 protein levels, synergizes with ABT-737, overcomes stroma-mediated drug resistance of the tumor microenvironment, decreased toxicity to bone marrow cells compared to daunorubicin, decreases tumor volume in xenograft model.	Proteasomal degradation of Mcl-1, caspase 3 activation, PARP cleavage	Sensitive AML patient samples = 7.2–8.8 μM U937 cells = 1.4 μM HL60 cells harboring ABT-737 resistance = 1.7 μM	10-fold less toxic to mouse bone marrow cells than ABT-737 and daunorubicin Less toxic to mouse hematopoietic progenitor cells compared to daunorubicin Maximum tolerated dose and 50% lethal dose of 20 mg/kg/day and 25 mg/kg/day respectively	[46]
	Triple negative breast cancer (MDA-MB-468)	Induces apoptosis	Decreased Mcl-1 protein levels, caspase 3 activation	MDA-MB-468 cells = 2 μM	ND	[65]
	Non-small cell lung cancer (H23, H460, H1299)	Induces apoptosis in cell-type specific manner, Mcl-1-dependent and -independent apoptosis, moderate increase in apoptosis with co-treatment with navitoclax (ABT-263),	Loss of Mcl-1 protein, PARP cleavage, PS externalization, release of cytochrome c, loss of mitochondrial membrane potential, mitochondrial fragmentation, mitochondrial ROS	ND	ND	[54]
	Myeloma (SKMM2, XG11, KMS-12PE, XG5, OPM2, NAN1, LP1, XG1, JJN-3, NAN10, MDN, U266, NAN3, L363, BCN, XG7, NCI-H929, NAN8, XG6, MM1-S)	Efficacy correlated with increased Mcl-1 and decreased Bcl-2, and wild type TP53	cleavage of caspase 3 and caspase 9, decreased Mcl-1 protein levels,	MM1-S cells = 0.7 μM XG6 cells = 1 μM NAN8 cells = 1.2 μM	ND	[64]
	Renal carcinoma (Caki, ACHN, A498), lung cancer (A549), hepatocellular carcinoma (SK-Hep1)	Marinopyrrole A sensitizes cells to TRAIL-induced apoptosis	miR-708 mediated cFLIP downregulation, CHOP-mediated DR5 upregulation, cleaved PARP, chromatin fragmentation, increased sub-G1%,	ND	ND	[59]
	Nasopharyngeal carcinoma (HK1, C666-1)	Inhibits cell proliferation, synergistic efficacy of marinopyrrole A + navitoclax (ABT-263)	Reduced expression level of Mcl-1 in both cell lines tested, modestly reduced expression level of Bcl-XL in HK1 cells and reduced Bcl-2 expression in C666-1 cells,	ND	ND	[44]
	Non-small cell lung cancer (H23, A549, H358, H460, H1299, H1650, H1755, H1975, H441)	Induced apoptosis, synergistic combination of marinopyrrole A + navitoclax (ABT-263) or ABT-737, AKT phosphorylation and EGFR status correlate with combination treatment susceptibility	Decreased Mcl-1 protein levels dependent on proteasomal degradation, cleaved PARP and caspase 3, increased PS externalization	H23 cells = 1.1 μM H441 cells = 9.2 μM	ND	[45]
	Melanoma (UACC903, A375M, 1205LU)	Induction of apoptosis, synergistic interaction of marinopyrrole A andABT-737, suppression of melanoma colony formation, inhibits growth of 3D melanoma spheroids	Down regulation of Mcl-1 protein through proteasomal degradation, increased PS externalization, PARP and caspase 3 cleavage, induction of BAX and BAK expression,	UACC903 cells = 2.8 μM A375M cells = 2.2 μM 1205Lu cells = 2.6 μM	ND	[36]
Marinopyrrole B 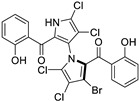	Colon cancer (HCT-116)	Cytotoxic	ND	HCT-116 cells = 9.0 μM	ND	[15,16]
Marinopyrrole C 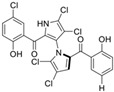	Colon cancer (HCT-116)	Cytotoxic	ND	HCT-116 cells = 0.4 μM	ND	[16]
Marinopyrrole F 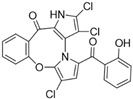	Colon cancer (HCT-116)	Cytotoxic	ND	HCT-116 cells = 6.1 μM	ND	[16]
Pyrrolomycin F1 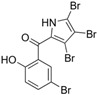	Colon cancer (HCT-116), Breast cancer (MCF7)	Inhibition of cell proliferation, induction of non-conventional cell death, possible induction of autophagy	Upregulation of acidic vesicular organelles, downregulation of AKT, increased ROS	HCT-116 cells = 0.35 μM MCF7 Cells = 0.61 μM	hTERT RPE-1/HCT-116 selectivity index = 179 hTERT RPE-1/MCF7 selectivity index = 103	[21]
Pyrrolomycin F2a 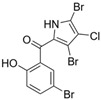	Colon cancer (HCT-116), Breast cancer (MCF7)	Inhibition of cell proliferation, induction of non-conventional cell death, possible induction of autophagy	Upregulation of acidic vesicular organelles, downregulation of AKT, increased ROS	HCT-116 cells = 0.49 μM MCF7 Cells = 0.73 μM	hTERT RPE-1/HCT-116 selectivity index = 177 hTERT RPE-1/MCF7 selectivity index = 119	[21]
Pyrrolomycin F2b 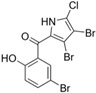	Colon cancer (HCT-116), Breast cancer (MCF7)	Inhibition of cell proliferation, induction of non-conventional cell death, possible induction of autophagy	Upregulation of acidic vesicular organelles, downregulation of AKT, increased ROS	HCT-116 cells = 0.41 μM MCF7 Cells = 0.65 μM	hTERT RPE-1/HCT-116 selectivity index = 150 hTERT RPE-1/MCF7 selectivity index = 95	[21]
Pyrrolomycin F3 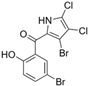	Colon cancer (HCT-116), Breast cancer (MCF7)	Inhibition of cell proliferation, induction of non-conventional cell death, possible induction of autophagy	Upregulation of acidic vesicular organelles, downregulation of AKT, increased ROS	HCT-116 cells = 0.88 μM MCF7 Cells = 1.21 μM	hTERT RPE-1/HCT-116 selectivity index = 199 hTERT RPE-1/MCF7 selectivity index = 145	[21]
Pyrrolomycin C 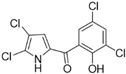	Colon cancer (HCT-116), Breast cancer (MCF7)	Induction of apoptosis	ND	HCT-116 cells = 0.8 μM MCF7 cells = 1.5 μM	hTERT RPE-1/HCT-116 selectivity index = 10 hTERT RPE-1/MCF7 selectivity index = 6	[27]

Natural marinopyrroles and pyrrolomycins that have been evaluated for cancer cell cytotoxicity and anticancer properties. ND = Not Determined.

**Table 2:** Synthetic Derivatives.

Compound	Host Cell Target	Bioactivity	Mechanism of Action	Cancer Cell IC_50_ (μM)	Mcl-1/Bim IC_50_ (μM)	Bcl-XL/Bim IC_50_ (μM)	ClogP/LogP	Toxicity to Healthy Cells	Reference
Cheng et al. Compound **9** 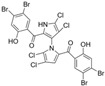	ND	ND	ND	ND	4.5 μM	7.3 μM	6.7	ND	[50]
Cheng et al. Compound **3** 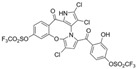	ND	ND	ND	ND	1.4 μM	2.3 μM	7.0	ND	[50]
Cheng et al. Compound **4** 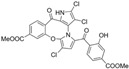	ND	ND	ND	ND	4.3 μM	3.4 μM	4.7	ND	[50]
Cheng et al. Compound 3 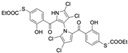	Triple negative breast cancer (MDA-MB-468)	Induces apoptosis, inhibition of tumor growth	Decrease in Mcl-1 protein level, induction of caspase 3 cleavage	3 μM	1.8 μM	1.2 μM	6.1	ND	[65]
Cheng et al. Compound **4** 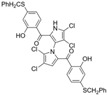	Triple negative breast cancer (MDA-MB-468)	Induces apoptosis, inhibition of tumor growth	Decrease in Mcl-1 protein level, induction of caspase 3 cleavage	28 μM	0.7 μM	0.6 μM	10.2	ND	[65]
Cheng et al. Compound **5** 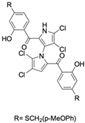	Triple negative breast cancer (MDA-MB-468)	Induces apoptosis, inhibition of tumor growth	Decrease in Mcl-1 protein level, induction of caspase 3 cleavage	50 μM	0.7 μM	0.6 μM	9.7	ND	[65]
Cheng et al. Compound **9** 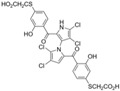	Triple negative breast cancer (MDA-MB-468)	Weakly induces apoptosis, inhibition of tumor growth	Decrease in Mcl-1 protein level, induction of caspase 3 cleavage	29 μM	6.1 μM	>100 μM	5.3	ND	[65]
Cheng et al. Compound **11** 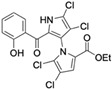	Triple negative breast cancer (MDA-MB-468)	Induces apoptosis, inhibition of tumor growth	Decrease in Mcl-1 protein level, induction of caspase 3 cleavage	2 μM	25.1 μM	96.6 μM	4.5	ND	[65]
Cheng et al. Compound **12** 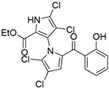	Triple negative breast cancer (MDA-MB-468)	Weakly induces apoptosis, inhibition of tumor growth	Decrease in Mcl-1 protein level, induction of caspase 3 cleavage	16 μM	11.5 μM	17.6 μM	4.5	ND	[65]
Li et al. Compound **32** 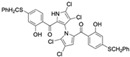	Triple negative breast cancer (MDA-MB-468)	Limited activity to induce apoptosis	ND	ND	0.7 μM	0.6 μM	NP	ND	[76]
Li et al. Compound **33** 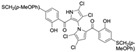	Triple negative breast cancer (MDA-MB-468)	Limited activity to induce apoptosis	ND	ND	0.7 μM	0.6 μM	NP	ND	[76]
Li et al. Compound **34** 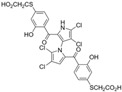	Triple negative breast cancer (MDA-MB-468)	Inactive at inducing apoptosis	ND	ND	6.1 μM	>100 μM	NP	ND	[76]
Li et al. Compound **36** 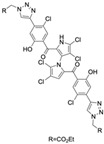	Triple negative breast cancer (MDA-MB-468)	Inactive at inducing apoptosis	ND	ND	7.8 μM	>100 μM	NP	ND	[76]
Li et al. Compound **37** 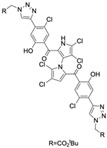	Triple negative breast cancer (MDA-MB-468)	Limited activity to induce apoptosis	ND	ND	1.6 μM	14.0 μM	NP	ND	[76]
Li et al. Compound **42** 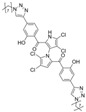	Triple negative breast cancer (MDA-MB-468)	Limited activity to induce apoptosis	ND	ND	0.6 μM	0.5 μM	NP	ND	[76]
Li et al. Compound **23** 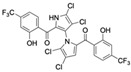	Triple negative breast cancer (MDA-MB-468)	Induces apoptosis	Decrease in Mcl-1 protein level, induction of caspase 3 cleavage	ND	8.1 μM	9.7 μM	NP	ND	[76]
Li et al. Compound **25** 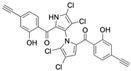	Triple negative breast cancer (MDA-MB-468)	Induces apoptosis	Decrease in Mcl-1 protein level, induction of caspase 3 cleavage	ND	3.9 μM	5.6 μM	NP	ND	[76]
Li et al. Compound **26** 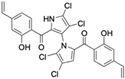	Triple negative breast cancer (MDA-MB-468)	Induces apoptosis	Decrease in Mcl-1 protein level, induction of caspase 3 cleavage	ND	3.7 μM	3.5 μM	NP	ND	[76]
Li et al. Compound **24** 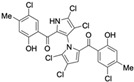	Triple negative breast cancer (MDA-MB-468)	Induces apoptosis	Decrease in Mcl-1 protein level, induction of caspase 3 cleavage	ND	2.6 μM	2.5 μM	NP	ND	[76]
Li et al. Compound **27** 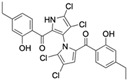	Triple negative breast cancer (MDA-MB-468)	Induces apoptosis	Decrease in Mcl-1 protein level, induction of caspase 3 cleavage	ND	2.1 μM	3.9 μM	NP	ND	[76]
Li et al. Compound **28** 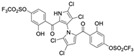	Triple negative breast cancer (MDA-MB-468)	Induces apoptosis	Decrease in Mcl-1 protein level, induction of caspase 3 cleavage	ND	1.0 μM	2.1 μM	NP	ND	[76]
Li et al. Compound **49** 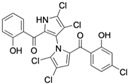	Triple negative breast cancer (MDA-MB-468)	Induces apoptosis	Decrease in Mcl-1 protein level, induction of caspase 3 cleavage	ND	6.5 μM	9.2 μM	NP	ND	[76]
Li et al. Compound **50** 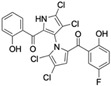	Triple negative breast cancer (MDA-MB-468)	Induces apoptosis	Decrease in Mcl-1 protein level, induction of caspase 3 cleavage	ND	8.9 μM	13.3 μM	NP	ND	[76]
Li et al. Compound **51** 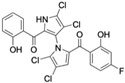	Triple negative breast cancer (MDA-MB-468)	Induces apoptosis	Decrease in Mcl-1 protein level, induction of caspase 3 cleavage	ND	9.6 μM	21.3 μM	NP	ND	[76]
McGuire et al. MP1 NP	Neuroblastoma (BE-2c, CCL-127), medulloblastoma (Daoy, ONS-76, D-283, D-341, HD-MB03)	Induces apoptosis/necrosis /autophagy /quiescent, synergy with co-treatment of MP1 + TEM, MP1 susceptibility correlated to MYCN amplification status, reduced tumor growth in vivo	Concentration-dependent decline in S-phase and increase in G2-phase, uncoupling of OXPHOS, inhibition of glycolysis, decrease in Mcl-1 protein, decrease in MYCN, increase in LC3I/II	BE-2c cells = 0.096 μM CCL-127 cells = 0.89 μM D-341 cells = 0.177 μM	ND	ND	3.8	MP1 tumor concentrations observed above IC_50_ MP1 was well tolerated in vivo No bone marrow toxicity observed.	[28,30]
Lotfy et al. Compound **5i** 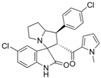	Triple negative breast cancer (MDA-MB-468), hepatocellular carcinoma (HepG2), colorectal adenocarcinoma (Cacco-2)	Induces apoptosis	PS externalization, increased p53, Bcl-2 down-regulated gene expression, caspase 3/7 activation, MDM2 inhibition	MDA-MB-468 cells = 0.2529 μM HepG2 cells = 1.3954 μM Caco-2 cells = 0.9079 μM	ND	ND	3.45	Therapeutic window between cancer cell potency and toxicity to normal lung fibroblasts (Wi-38)	[77]
Lotfy et al. Compound **5q** 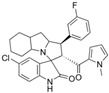	Triple negative breast cancer (MDA-MB-468), hepatocellular carcinoma (HepG2), colorectal adenocarcinoma (Cacco-2)	Induces apoptosis	PS externalization, increased p53, Bcl-2 down-regulated gene expression, caspase 3/7 activation, MDM2 inhibition	MDA-MB-468 cells = 0.0002 μM HepG2 cells = 0.0091 μM Caco-2 cells = 0.0003 μM	ND	ND	4.13	Therapeutic window between cancer cell potency and toxicity to normal lung fibroblasts (Wi-38)	[77]
Hughes et al. Compound **11** 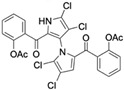	Colon cancer (HCT-116)	Cytotoxic	ND	HCT-116 cells = 0.42 ug/mL	ND	ND	NP	ND	[16]
Hughes et al. Compound **14** 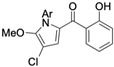	Colon cancer (HCT-116)	Cytotoxic	ND	HCT-116 cells = 1.1 ug/mL	ND	ND	NP	ND	[16]
Hughes et al. Compound **16** 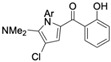	Colon cancer (HCT-116)	Cytotoxic	ND	HCT-116 cells = 4.4 ug/mL	ND	ND	NP	ND	[16]
Raimondi et al. Compound **1** 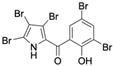	Colon cancer (HCT-116), breast cancer (MCF7)	Induces necrosis, inhibits cancer cell proliferation	Morphological changes including membrane elongations resembling filopodia	HCT-116 cells = 1.3 μM MCF7 cells = 1.2 μM	ND	ND	4.889	HCT-116/hTERT RPE-1 selectivity index = 44 MCF7/hTERT RPE-1 selectivity index = 47	[21,27]
Raimondi et al. Compound **5a** 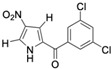	Colon cancer (HCT-116), breast cancer (MCF7)	Induces apoptosis	Morphological changes including membrane elongations resembling filopodia	HCT-116 cells = 1.9 μM MCF7 cells = 2.2 μM	ND	ND	2.496	HCT-116/hTERT RPE-1 selectivity index = 35 MCF7/hTERT RPE-1 selectivity index = 30	[27]
Raimondi et al. Compound **5d** 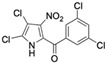	Colon cancer (HCT-116), breast cancer (MCF7)	Induces apoptosis	Morphological changes including membrane elongations resembling filopodia	HCT-116 cells = 1.6 μM MCF7 cells = 1.6 μM	ND	ND	3.746	HCT-116/hTERT RPE-1 selectivity index = 143 MCF7/hTERT RPE-1 selectivity index = 143	[27]
Doi et al. KS04 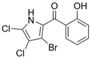	Monocytic leukemia (U937), chronic myelogenous leukemia (K562), acute T-cell leukemia (Jurkat, JurkatΔBak), acute myeloid leukemia (HL60, Kasumi-1, THP-1), murine acute myeloid leukemia (C1498), multiple myeloma (RPMI 8226, MM.1S, NCI-H929, U266)	Induces apoptosis in Mcl-1-dependent cancer cells, synergistic interaction between KS04 + ABT-737, overcomes stroma-mediated drug resistance	Induces caspase 3 and PARP cleavage, decreased Mcl-1 protein half-life	NCI-H929 cells = 1.00 μM C1498 cells = 1.12 μM RPMI 8226 cells = 1.87 μM	ND	ND	NP	Less toxic to primary mouse bone marrow cells compared to daunorubicin and ABT-737	[35]
Doi et al. KS18 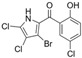	Monocytic leukemia (U937), chronic myelogenous leukemia (K562), acute T-cell leukemia (Jurkat, JurkatΔBak), acute myeloid leukemia (HL60, Kasumi-1, THP-1), murine acute myeloid leukemia (C1498), multiple myeloma (RPMI 8226, MM.1S, NCI-H929, U266)	Induces apoptosis in Mcl-1-dependent cancer cells, synergistic interaction between KS18 + ABT-737, synergistic interaction between KS18 + bortezomib, overcomes stroma-mediated drug resistance, decreases tumor volume in vivo	Induces caspase 3 and PARP cleavage, decreased Mcl-1 protein half-life, modulation STAT-3 activation	U266 cells = 0.9 μM MM.1S cells = 0.79 μM U937 cells = 0.5 μM	ND	ND	NP	Less toxic to primary mouse bone marrow cells compared to daunorubicin and ABT-737 Intraperitoneal maximum tolerated dose and median lethal dose of 10 mg/kg/day and 15 mg/kg/day Oral maximum tolerated dose and median lethal dose of 20 mg/kg/day and 30 mg/kg/day	[35,78]

Some of the synthetic derivatives of marinopyrroles and pyrrolomycins that have been evaluated for cancer cell cytotoxicity and anticancer properties. ND = Not Determined. NP = Not Provided.

## Data Availability

No new data were created or analyzed in this study. Data sharing is not applicable to this article.
